# Characteristics Research of a High Sensitivity Piezoelectric MOSFET Acceleration Sensor

**DOI:** 10.3390/s20174988

**Published:** 2020-09-03

**Authors:** Chunpeng Ai, Xiaofeng Zhao, Dianzhong Wen

**Affiliations:** Key Laboratory of Electronics Engineering College of Heilongjiang Province, Heilongjiang University, Harbin 150006, China; aichunpeng@hlju.edu.cn (C.A.); zhaoxiaofeng@hlju.edu.cn (X.Z.)

**Keywords:** piezoelectric MOSFET, piezoelectric beam, acceleration sensor, high sensitivity

## Abstract

In order to improve the output sensitivity of the piezoelectric acceleration sensor, this paper proposed a high sensitivity acceleration sensor based on a piezoelectric metal oxide semiconductor field effect transistor (MOSFET). It is constituted by a piezoelectric beam and an N-channel depletion MOSFET. A silicon cantilever beam with Pt/ZnO/Pt/Ti multilayer structure is used as a piezoelectric beam. Based on the piezoelectric effect, the piezoelectric beam generates charges when it is subjected to acceleration. Due to the large input impedance of the MOSFET, the charge generated by the piezoelectric beam can be used as a gate control signal to achieve the purpose of converting the output charge of the piezoelectric beam into current. The test results show that when the external excitation acceleration increases from 0.2 g to 1.5 g with an increment of 0.1 g, the peak-to-peak value of the output voltage of the proposed sensors increases from 0.327 V to 2.774 V at a frequency of 1075 Hz. The voltage sensitivity of the piezoelectric beam is 0.85 V/g and that of the proposed acceleration sensor was 2.05 V/g, which is 2.41 times higher than the piezoelectric beam. The proposed sensor can effectively improve the voltage output sensitivity and can be used in the field of structural health monitoring.

## 1. Introduction

Micro-electro-mechanical system (MEMS) acceleration sensors, with the advantages of low cost, low power consumption, high compatibility with integrated circuit (IC) process and high integration [1,2,3,4], have a wide range of applications in automotive electronics, structural health monitoring, navigation and other fields [5,6,7,8]. MEMS acceleration sensors usually include piezoresistive [9,10], capacitive [11] and piezoelectric acceleration sensors [12,13]. Piezoresistive accelerometer usually consists of a deformable structure and varistors. Under the action of external acceleration, the deformation of the deformable structure causes the resistance of the varistor to change, thereby measuring the acceleration. The varistors are usually made into a Wheatstone bridge structure to improve the output sensitivity. The piezoresistive accelerometer has the advantages of good stability, wide measurement range, and is also limited by ambient temperature [14,15,16]. A capacitive acceleration sensor is composed of fixed plates and movable plates. The gap or area of the plate capacitor changes while the external acceleration is applied. The applied acceleration is obtained by measuring the change of capacitance. High sensitivity and zero frequency response are its advantages, while its disadvantages are high impedance and nonlinearity [17,18,19]. The working principle of the piezoelectric acceleration sensor is similar to that of the piezoresistive type, but the difference is that the piezoresistive material is replaced by piezoelectric material. Compared with piezoresistive and capacitive MEMS acceleration sensors, piezoelectric MEMS acceleration sensors have advantages of low power cost and high range of applying frequency; at the same time, they are also limited by high output impedance, weak output signal and so on [20,21,22,23].

The researchers are committed to improving the structure of the piezoelectric acceleration sensor so as to improve its performance, especially its sensitivity. For example, Jin Xie et al. present a MEMS piezoelectric in-plane resonant accelerometer with a two-stage microleverage mechanism. The sensitivity of the device is 28.4 Hz/g and the relative sensitivity is 201 ppm/g at the base frequency around 140.7 kHz, which are 57% and 268% higher than previously reported data [24]. Qiang Zou et al. reported novel single- and tri-axis piezoelectric-bimorph accelerometers that are built on parylene beams with ZnO thin films. A highly symmetric quad-beam bimorph structure with a single proof mass is used for tri-axis acceleration sensing. The unamplified sensitivities of the *x*-axis, *y*-axis, and *z*-axis are 0.93, 1.13, and 0.88 mV/g, respectively [13]. At the same time, the researchers also studied the doped piezoelectric materials in order to improve the piezoelectric properties so as to improve the sensitivity. Ramany et al. presented a nano-electro-mechanical systems accelerometer using undoped zinc oxide nanorods and 1 wt. (Weight) %, 3 wt.% and 5 wt.% of vanadium-doped zinc oxide nanorods as an active layer. The highest sensitivity of 3.528 V/g was acquired for 5 wt.% of vanadium-doped zinc oxide with maximum output voltages of 2.30 V and 2.9 V at 9 Hz resonant frequency and 1 g acceleration, respectively [25].

In this work, by taking advantage of the high gate sensitivity of MOSFET, in order to improve the output sensitivity and reduce the output impedance of piezoelectric acceleration sensors, we designed a piezoelectric MOSFET acceleration sensor (PMAS) structure. The PMAS with high sensitivity can be used in acceleration monitoring under special frequency vibration environments, such as health monitoring on turning tools.

## 2. Basic Structure and Operating Principle

### 2.1. Basic Structure

**Figure 1:**
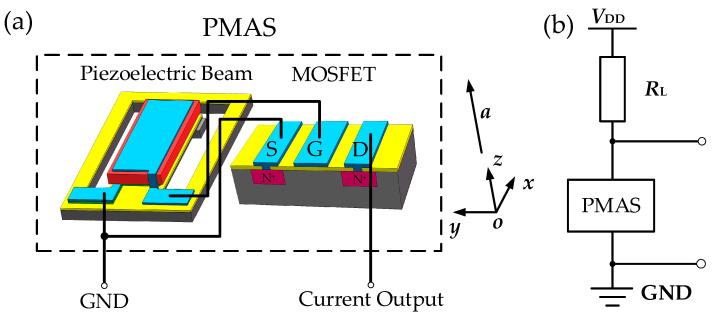
Basic structure and test circuit of piezoelectric metal oxide semiconductor field effect transistor (MOSFET) acceleration sensor (PMAS): (**a**) basic structure; (**b**) test circuit.

The structure of PMAS is shown in Figure 1a. It consists of a piezoelectric beam and an N-channel depletion MOSFET. The piezoelectric beam is a two-ended device: one end is connected with the source of MOSFET as the ground terminal of PMAS, the other end is connected with the gate of MOSFET to control the output current of MOSFET, and the drain of MOSFET is the current output terminal of PMAS. The direction of the measured acceleration is parallel to the *z*-axis as shown in Figure 1a. The acceleration applied by the vibration system is reciprocating up and down along the *z*-axis. Therefore, the output signal of the piezoelectric beam is a sinusoidal signal with a certain frequency. The N-channel depletion MOSFET is chosen to ensure that the MOSFET works in the triode region. As shown in Figure 1b, a load resistance *R*_L_ is used in series with PMAS to convert the current signal into a voltage signal for output.

**Figure 2:**
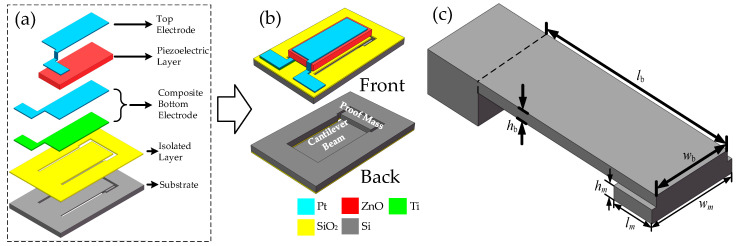
Basic structure of piezoelectric beam: (**a**) explosion view; (**b**) front and back side, (**c**) dimension of the cantilever beam.

The structure of the piezoelectric beam is shown in Figure 2a, which consists of a silicon cantilever beam substrate with a proof mass and a piezoelectric multilayer structure. Silicon cantilever beam substrate is fabricated by lithography and inductive coupled plasma (ICP) etching technology. The piezoelectric multilayer structure, including electrodes (Pt top electrodes and Pt/Ti composite bottom electrode) and a ZnO piezoelectric layer, was deposited by direct-current (DC) and radio frequency (RF) magnetron sputtering under the optimized parameters [26], respectively. Then, 5 wt% Li-doped ZnO was used as the piezoelectric layer. In general, ZnO is an n-type semiconductor due to the defects of oxygen vacancy and zinc interstitial atoms in the process of fabrication. After doping lithium as an acceptor impurity, the resistivity of ZnO is increased, thereby enhancing its piezoelectric properties. The piezoelectric beam is designed to be 9800 × 5800 × 500 μm^3^ in size. Figure 2c shows the dimension of the cantilever beam. *l*_b_, *w*_b_ and *h*_b_ are the length, width and height of the cantilever beam, respectively. *l*_m_, *w*_m_ and *h*_m_ are the length, width and height of the proof mass, respectively. *l*_b_ × *w*_b_ × *h*_b_ was designed to be 6000 × 2400 × 80 μm^3^, *l*_m_ × *w*_m_ × *h*_m_ was designed to be 1000 × 2700 × 395 μm^3^. After the piezoelectric beam was manufactured, it was rigidly pasted on the customized test printed circuit board (PCB), and the piezoelectric beam’s electrodes were connected with the PCB plate electrodes by a chip press welder (KNS4526, Kullicke & Soffa, Haifa, Israel).

### 2.2. Operating Principle

**Figure 3:**
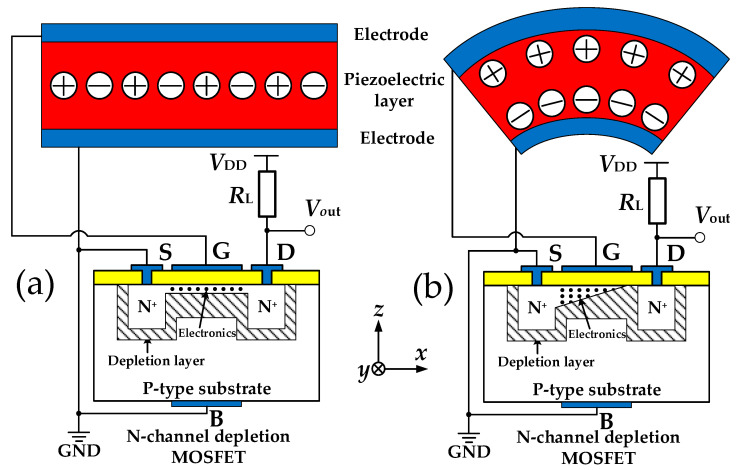
Working principle of piezoelectric PMAS: (**a**) without external acceleration; (**b**) with external acceleration.

As shown in Figure 3a, the piezoelectric beam does not deform without external force. The centers of positive and negative charges in the piezoelectric layer coincide with each other; the whole piezoelectric layer is electrically neutral and there are no charge outputs. The PMAS also has a certain output when it is not affected by acceleration due to the existence of a conductive channel in the N-channel depletion MOSFET even when the gate voltage is 0. When external acceleration is applied, according to Newton’s first and second laws, the proof mass will produce forces opposite to the acceleration direction due to the inertia, which can cause the piezoelectric beam to be deformed. At this time, the bending creates the electric dipole moment in the piezoelectric layer, forcing the positive and negative charge centers to separate. The same amount of charges of different signs are generated on the upper and lower surfaces of the piezoelectric layer. The generated charge is used as the gate signal of the MOSFET, which can directly control the width of the channel, thereby controlling the drain current of the MOSFET.

**Figure 4:**
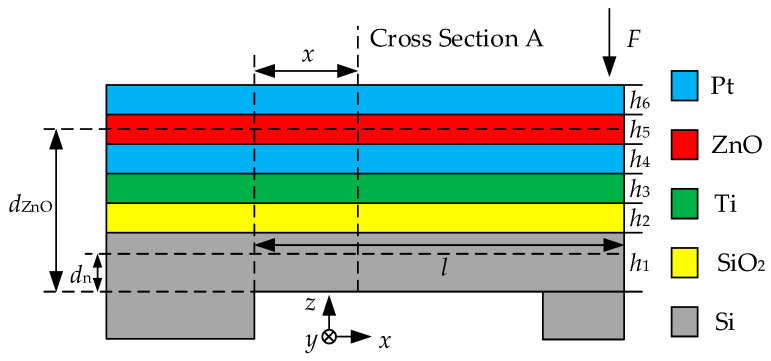
Stress analysis diagram of piezoelectric beam structure.

The stress analysis of the piezoelectric beam is shown in Figure 4. *d*_ZnO_ is the distance from the ZnO thin film to the bottom of the Si substrate, *d*_n_ is the distance from the neutral plane of the multilayer structure to the bottom of the Si substrate, *h*_1_, *h*_2_, …, *h*_6_ represent the thickness of Si, SiO_2_, Ti, Pt, ZnO and Pt layers, respectively.

The main assumptions are as follows: (1) since the length and width are far greater than the thickness of the cantilever beam, it is considered that the cantilever beam has pure bending deformation and ignores the shear stress; (2) the beam bending caused by the residual stress is ignored; (3) the beam bending caused by the residual stress is ignored; (4) it is assumed that there is no relative sliding between each thin films; (5) the cantilever beam is in an open environment and there are no upper and lower fixed plates, so the influence of air damping is ignored [27,28,29].

According to the Euler-Bernoulli’s equation, when the free end of the cantilever beam is subjected to a concentrated transverse force *F*, the stress on the cross section A of ZnO thin film is:

(1) $$\sigma_{A}=\frac{M_{A}}{I}\left|d_{\mathrm{ZnO}}-d_{n}\right|=\frac{F\cdot x}{I}\left|d_{\mathrm{ZnO}}-d_{n}\right|$$

where *M*_A_ is the bending moment, *I* is the moment of inertia of the multilayer structure.

The equivalent width of layer *i* relative to Si substrate is:

(2) $$w_{{}_{i}}^{\prime }=\frac{E_{i}}{E_{\mathrm{Si}}}w_{\mathrm{Si}},\;i=1,2,\ldots ,6$$

where *w^′^_i_* is the width of each thin film, *E_i_* is the young’s modulus, *i* represents Si, SiO_2_, Ti, Pt, ZnO and Pt layers, respectively.

Therefore, the equivalent sectional area *S*_i_ can be expressed as:

(3) $$S_{i}=w_{i}\cdot h_{i},\;i=1,2,\ldots ,6$$

The equivalent second moment of inertia of layer *i* is:

(4) $$I_{i}^{\prime }=\frac{h_{i}^{3}}{12}w_{i},\;i=1,2,\ldots ,6$$

The distance from the *i* layer to the bottom of Si substrate is:

(5) $$z_{i}=\sum_{i=1}^{6}h_{i}-\frac{h_{i}}{2}$$

The distance from the neutral plane of the multilayer structure to the Si substrate is:

(6) $$z^{\prime }=\frac{\sum_{i=1}^{6}S_{i}d_{i},}{\sum_{i=1}^{6}S_{i}}$$

The second moment of inertia is:

(7) $$I^{\prime }=\sum_{i=1}^{6}\left[I_{i}+S_{i}{\left(z_{i}-z^{\prime }\right)}^{2}\right]$$

Substituting Equations (6) and (7) into (1), the cross-section stress of ZnO is:

(8) $$\sigma =\frac{F\cdot x\cdot \left|d_{\mathrm{ZnO}}-d_{n}\right|}{\sum_{i=1}^{6}\left\{\frac{h_{i}^{3}w_{i}}{12}+S_{i}\left[\left(\sum_{i=1}^{6}h_{i}-\frac{h_{i}}{2}\right)-\frac{\sum_{i=1}^{6}S_{i}d_{i},}{\sum_{i=1}^{6}S_{i}}\right]\right\}},\;0\leq x\leq l$$

According to the Hooke’s law and the theories in mechanics of materials, a deflection *δ* will appear when a force *F* is applied at the free end of the cantilever beam, and the deflection *δ* of the free end can be expressed as [30,31]:

(9) $$\delta =\frac{F}{k}=\frac{Fl_{b}{}^{3}}{3EI}=\frac{4Fl_{b}{}^{3}}{Ew_{b}h_{b}^{3}}$$

where *k* is the stiffness of the cantilever beam.

The fundamental resonant frequency is [32]:

(10) $$f=\frac{\alpha_{n}^{2}}{2\pi }\sqrt{\frac{EI}{mL^{4}}}$$

where *E* is the modulus of elasticity, *I* is the area moment of inertia, *α*_n_ is a constant which its value depends on the mode of cantilever beam’s vibration, *m* is the mass of the cantilever beam, *L* is the length of the cantilever beam.

Considering the influence of the mass proof on the resonant frequency of cantilever beam, the first mode resonant frequency of the cantilever beam can be expressed as [33,34,35]:

(11) $$f={\frac{1.875}{2\pi }}^{2}\sqrt{\frac{0.236EI}{{\left(l_{b}-l_{m}/2\right)}^{3}\left(0.236\rho h_{b}w_{b}\left(l_{b}-\frac{l_{m}}{2}\right)+\rho h_{b}w_{b}\frac{l_{m}}{2}+\rho h_{m}w_{m}l_{m}\right)}}$$

where *ρ* is the density of Si. The moment of inertia of multilayer structure is ignored due to the thickness of the multilayer structure being much less than that of the cantilever beam.

It can be seen that *l_c_* is inversely proportional to the *f* frequency of the cantilever beam, and the change of *l_c_* has a greater impact on resonant frequency. Based on the piezoelectric effect, the surface charge density of ZnO is [26]:

(12) $$D_{3}=d_{31}\sigma$$

Therefore, under the external acceleration, the charges generated by the piezoelectric beam are:

(13) $$\begin{array}{ll} q & =\int_{0}^{w_{b}}\int_{0}^{l_{b}}D_{3}dxdy\\ & =\int_{0}^{w_{b}}\int_{0}^{l_{b}}d_{31}\sigma dxdy\\ & =\int_{0}^{w_{b}}\int_{0}^{l_{b}}\frac{d_{31}Fx\left|d_{\mathrm{ZnO}}-d_{n}\right|}{\sum_{i=1}^{6}\left\{\frac{h_{i}^{3}w_{i}}{12}+S_{i}\left[\left(\sum_{i=1}^{6}h_{i}-\frac{h_{i}}{2}\right)-\frac{\sum_{i=1}^{6}S_{i}d_{i},}{\sum_{i=1}^{6}S_{i}}\right]\right\}}dxdy\\ & =\frac{w_{b}l_{b}^{2}d_{31}F\left|d_{\mathrm{ZnO}}-d_{n}\right|}{2\sum_{i=1}^{6}\left\{\frac{h_{i}^{3}w_{i}}{12}+S_{i}\left[\left(\sum_{i=1}^{6}h_{i}-\frac{h_{i}}{2}\right)-\frac{\sum_{i=1}^{6}S_{i}d_{i},}{\sum_{i=1}^{6}S_{i}}\right]\right\}} \end{array}$$

The piezoelectric beam can be approximately considered as a parallel plate capacitor with a dielectric inside, so the output voltage *V*_B_ of the piezoelectric beam is:

(14) $$V_{B}=\frac{q}{C}$$

In this case, due to the MOSFET works in the triode region, the relationship between source drain current *I*_DS_ and gate voltage *V*_GS_ can be expressed as follows [36]:

(15) $$I_{\mathrm{DS}}=\frac{\mu_{n}WC_{\mathrm{ox}}}{L}\left[\left(V_{\mathrm{GS}}-V_{T}\right)V_{\mathrm{DS}}-\frac{1}{2}V_{\mathrm{DS}}^{2}\right]$$

where, *μ*_n_ is dependent effective mobility, *W* is the channel width, *L* is the effective channel length, *C*_ox_ is the insulation capacitance, *V*_GS_ is the gate voltage which is equal to *V*_B_*, V*_T_ is the threshold voltage. 

The output voltage of PMAS is:

(16) $$V_{\mathrm{out}}=V_{\mathrm{DD}}-V_{R}=V_{\mathrm{DD}}-I_{\mathrm{DS}}R_{L}$$

Therefore, when the piezoelectric beam is connected to the gate of MOSFET, the output voltage of the piezoelectric beam is equal to *V*_GS_. With Equations (13)–(16), the relationship between *V*_out_ and *F* is:

(17) $$V_{\mathrm{out}}=V_{\mathrm{DD}}-\frac{\mu_{n}WC_{\mathrm{ox}}}{L}\left[\left(\frac{q}{C}-V_{T}\right)V_{\mathrm{DS}}-\frac{1}{2}V_{\mathrm{DS}}^{2}\right]R_{L}$$

It can be seen from Equation (17) that the output voltage of the PMAS is proportional to the channel width-to-length ratio of the MOSFET.

## 3. Fabrication Technology

**Figure 5:**
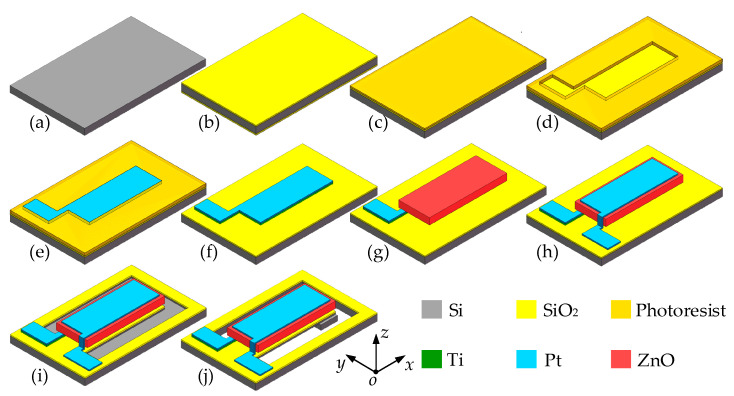
Fabrication process of the piezoelectric beam: (**a**) Si wafer; (**b**) growing SiO_2_; (**c**) coating photoresist; (**d**) patterning photoresist; (**e**) depositing Pt/Ti; (**f**) removing photoresist; (**g**) depositing ZnO; (**h**) depositing Pt; (**i**) etching on the top side; (**j**) releasing cantilever beam.

Figure 5 shows the fabrication process of the piezoelectric beam. The n-type <100> orientation silicon wafer was cleaned by the standard Radio Cooperation of America (RCA) process (Figure 5a), and the silicon dioxide layer was grown by the thermal oxidation method as the isolation layer (Figure 5b). In the manufacturing process of Pt/ZnO/Pt/Ti piezoelectric multilayer structure, the lift-off process is selected to complete the fabrication of the piezoelectric multilayer structure. As shown in Figure 5c–h, firstly, the photoresist is evenly coated on the surface of the substrate layer, then patterned by photolithography. The Pt/Ti composite electrode is coated by RF magnetron sputtering, and the photoresist is removed by the stripping solution to complete the fabrication of the bottom electrode. ZnO layer and top electrode were also prepared by the lift-off process as the Pt/Ti bottom electrode. After that, the cantilever structure is released by twice photolithography and inductively coupled plasma (ICP) etching to complete the fabrication of the piezoelectric beam (Figure 5i–j). In order to improve the piezoelectric properties of the ZnO piezoelectric layer, we doped ZnO with lithium. Li atoms with a small atomic radius as acceptor impurities can increase the resistivity of the ZnO piezoelectric thin film and increase the output impedance, thereby achieving the purpose of improving the piezoelectric properties of ZnO. During the preparation process, most of the doped lithium atoms will replace the positions of the zinc atoms and cause the decrease of the lattice constant. As a result, the residual stress in ZnO thin film is compressive stress [37].

## 4. Results and Discussion

### 4.1. Test System

**Figure 6:**
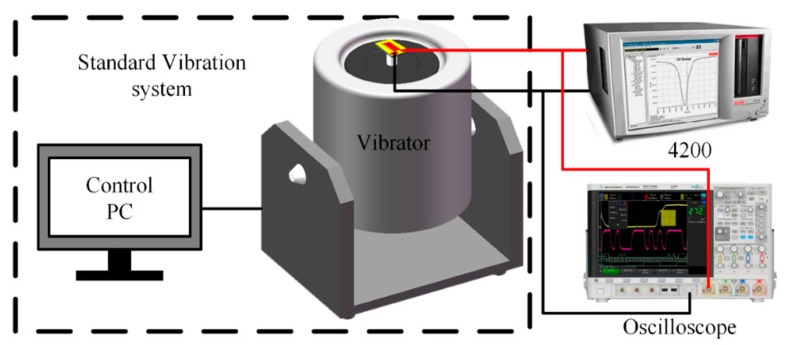
Testing system of acceleration sensor.

Figure 6 shows the test system for the proposed acceleration sensor. It consisted of a standard vibrator (Dongling ESS-050, Dongling Vibration Test Instrument Co., Ltd., Suzhou, China), an oscilloscope (DSO-X 4154A, Agilent Technologies Inc., Santa Clara, CA, USA), a semiconductor characteristic analysis system (4200SCS, KEITHLEY 4200, Keithley, Cleveland, OH, USA) and a control computer. The system can apply acceleration from 0 to 30 G; the lower limit of the frequency is 50 Hz, and the upper limit is 20,000 Hz.

### 4.2. Frequency Characteristic of the Piezoelectric Beam

**Figure 7:**
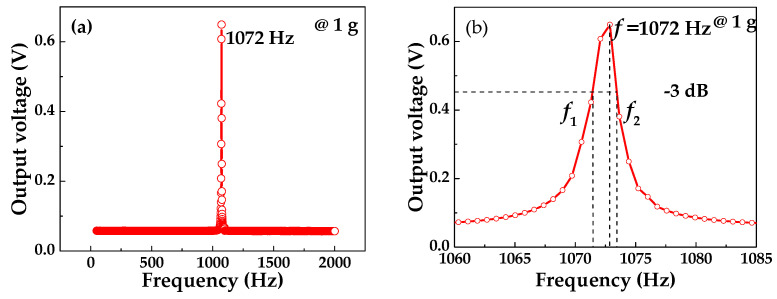
Relationship curve between output voltage and excitation frequency: (**a**) resonant frequency; (**b**) quality factor.

The frequency characteristic of the piezoelectric beam was analyzed by the sweep mode of the vibrator. The range of the excitation frequency was set from 20 to 2000 Hz, and applied acceleration was 1 g constant along the *z*-axis direction of the piezoelectric beam. The piezoelectric beam was rigidly connected with the vibration table by a customized fixture. When the excitation frequency reached a certain value, the output of the piezoelectric beam reached its maximum value for the first time. At this time, the excitation frequency was the first resonance frequency of the piezoelectric beam. Figure 7a shows the relationship between the output voltage and the excitation frequency of the piezoelectric beam. When the excitation frequency reached 1072 Hz, the output of the piezoelectric beam reached the maximum value of 0.649 V. There is only one peak within 50–2000 Hz, which proves that 1072 Hz is the first-order resonance frequency of the piezoelectric beam.

According to Figure 7a, the quality factor of the piezoelectric beam can be roughly analyzed as shown in Figure 7b. The quality factor *Q* represents the energy dissipated by the body in overcoming the internal friction in resonance.

(18) $$Q=2\pi \frac{E_{s}}{E_{c}}=\frac{f}{f_{1}-f_{2}}$$

where *E*_s_ is the mechanical energy stored by the oscillator in the resonant state and *E*_c_ is the energy dissipated by the oscillator in the resonant state every cycle, *f* is the resonance frequency and *f*_1_, *f*_2_ is the half power point frequency (−3 dB). From Figure 5b we can estimate that *Q* is 529.1.

Table 1 shows the design dimensions of piezoelectric beams. By substituting the data in Table 1 into Equation (10), the theoretical resonance frequency of the piezoelectric beam is 995 Hz, which is lower than the test result 1072 Hz. This is mainly due to the deviation of the thickness and length of the cantilever beam from the design value during the manufacturing process.

### 4.3. I_DS_-V_DS_ Characteristic of MOSFET with Piezoelectric Beam

**Figure 8:**
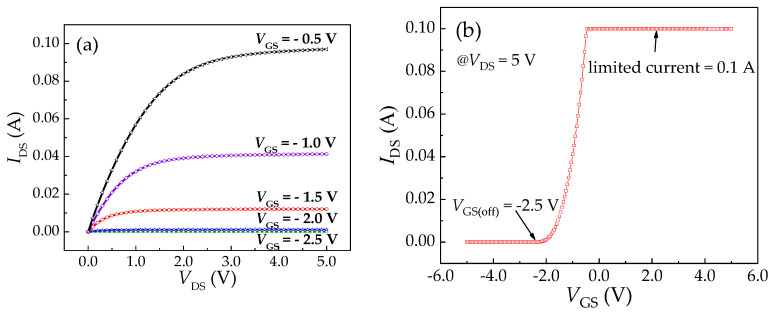
Characteristics curves of the MOSFET: (**a**) *I*_DS_*-V*_DS_ characteristics curves; (**b**) transition characteristic curve.

The *I-V* characteristic and transfer characteristic curves of the MOSFET were tested by 4200SCS, as shown in Figure 8a. Considering that the maximum limited current of 4200SCS is 0.1 A, the voltage range of *V*_GS_ was set from −2.5 V to −0.5 V with an increment of −0.5 V. Figure 8b shows the transition characteristic curve of MOSFET, *V*_DS_ is 5 V in testing. It can be concluded that the pinch-off voltage of MOSFET (*V*_GS(off)_) is −2.5 V; with the increase of *V*_GS_, the *I*_DS_ also increases. When *V*_GS_ reaches −0.45 V, the limit current of the test instrument 0.1 A is reached.

**Figure 9:**
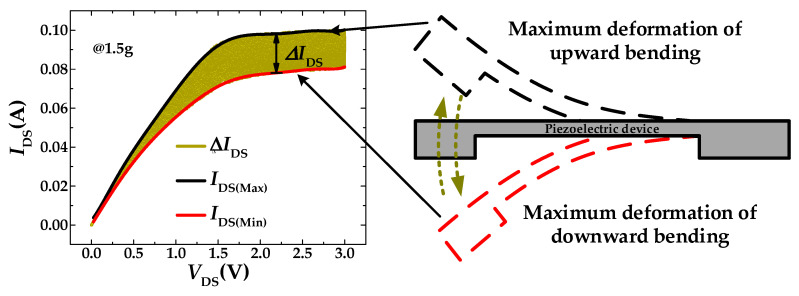
*I*_DS_*-V*_DS_ characteristics curves of the MOSFET with the piezoelectric beam.

The test circuit shown in Figure 1b, 4200SCS, is connected with two output terminals of PMAS for data acquisition. The output voltage of the piezoelectric beam was used as gate voltage of MOSFET. In order to ensure that the output voltage of the piezoelectric beam can reach the maximum value, the test frequency was set at 1072 Hz, which is the resonance frequency of the piezoelectric beam. The *I-V* characteristic curves of the MOSFET are shown in Figure 9 (*V*_DD_ = 5 V, *R*_L_ = 10 kΩ and applied acceleration was 1.5 g). Under the external acceleration generated by the stander vibration system, the proof mass drove the piezoelectric beam to vibrate up and down, which makes the *I*_DS_ of MOSFET fluctuate in a certain range. The maximum and minimum values of the wave range correspond to the maximum deformation of upward and downward bending of the piezoelectric beam, respectively. When *V*_DS_ is a certain value, the difference between the upper and the lower limit is Δ*I*_DS_. With the increase of external excitation acceleration, the vibration amplitude of the piezoelectric beam increases and further increases the width of the curves. That means Δ*I*_DS_ will increase with as acceleration increases. Because the signal produced by the piezoelectric beam is sinusoidal under the action of the stander vibration system, Δ*I*_DS_ can be used as an important parameter to measure the output sensitivity of PMAS. In the test circuit of Figure 1b, by selecting appropriate load resistance *R*_L_, PMAS can amplify the signal of the piezoelectric beam so as to improve the sensitivity of output voltage.

### 4.4. Sensitivity Characteristic of PMAS

**Figure 10:**
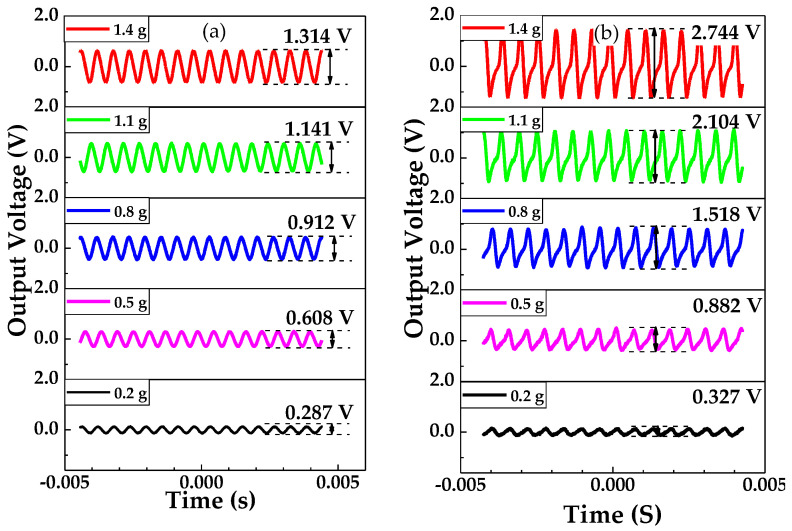
Output characteristics of the piezoelectric beam and PMA under the acceleration range from 0.2 g to 1.4 g: (**a**) output voltage curves of piezoelectric beam; (**b**) output voltage of PMAS.

Figure 10a shows the output voltage curves of the piezoelectric beam and PMAS, where the peak-to-peak value of the output sinusoidal curves are defined as output voltage *V*_piezo_ and *V*_PMAS_. *V*_piezo_ increased from 0.287 V to 1.314 V at the excitation frequency of 1072 Hz, and the acceleration range was from 0.2 to 1.4 g. It can be concluded that *V*_piezo_ increases with the increment of excitation acceleration, and the relationship between them is approximately linear. Figure 10b shows the output voltage curve of the PMAS (*V*_PMAS_). In the conditions of excitation frequency of 1072 Hz and applied acceleration range from 0.2 to 1.4 g, *V*_PMAS_ increased from 0.327 V to 2.744 V. Compared with *V*_piezo_, at the same condition *V*_PMAS_ increased significantly and *V*_PMAS_ was approximately linear with the excitation acceleration.

According to Equation (13), the theoretical output voltage of the piezoelectric beam is 2.110 V under the acceleration condition of 1 g, which is higher than the actual output voltage of 1.141 V. The reason is that the thickness of the cantilever beam deviates from the theoretical value due to the uniformity error of ICP etching. The thickness of the cantilever beam is not uniform, which leads to the uneven distribution of stress when the cantilever beam is forced to bend. At the same time, there will be defects inside the ZnO piezoelectric thin film during the manufacturing process, which causes the charge generated by the stress to be less than the theoretical value.

**Figure 11:**
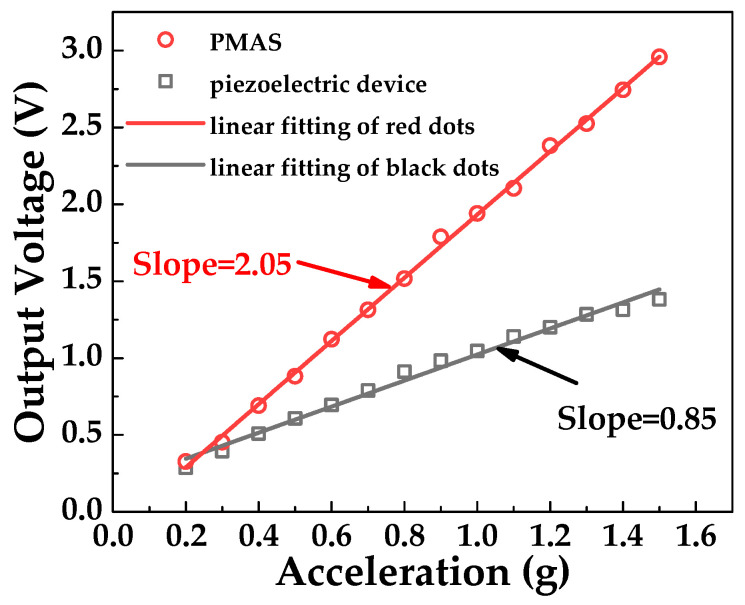
Relationship curves between output voltage and acceleration of PMAS and the piezoelectric beam.

Figure 11 shows the comparison of *V*_PMAS_ and *V*_piezo_ with the increase of acceleration. It can be seen that under the same external acceleration, the output voltage of PMAS is significantly higher than that of piezoelectric beam. It means that MOSFET in PMAS plays a role of amplification. After that, two groups of output curves are fitted linearly, and it can be concluded that the two groups of curves have good linearity and the slopes of the fitting curves are the output voltage sensitivity of the two devices. The sensitivity of PMAS is 2.05 V/g, which is 2.41 times higher than that of the piezoelectric beam at 0.85 V/g. It proved that the PMAS structure can effectively improve the sensitivity of the piezoelectric beam. The charge output of the piezoelectric beam as the gate control voltage of the MOSFET can effectively convert the output charge into an output current, and it can also increase the load capacity of the piezoelectric acceleration sensor. However, the proposed sensor has a relatively narrow frequency range, which limits its application field. In addition, it can be seen from Equation (14) that the width-to-length ratio of the MOSFET is directly proportional to the output current. The *I*_DS_ can be improved by adjusting the aspect ratio of the MOSFET during the manufacturing process, thereby further improving the output sensitivity of the PMAS.

Table 2 shows the performance comparison of piezoelectric acceleration sensors. The proposed sensor has certain advantages in sensitivity, but does not dominate in terms of chip size. It can also be seen from the comparison that piezoelectric acceleration sensors have higher sensitivity, but at a disadvantage in terms of measurement range and load capacity.

## 5. Conclusions

In summary, this paper proposed a high sensitivity piezoelectric acceleration sensor. It consisted of a piezoelectric beam and an N-channel depletion MOSFET. Utilizing the advantage of MOSFET’s high input impedance, the output signal of the piezoelectric beam was used to drive the MOSFET, thereby converting the output charge of the piezoelectric beam into output current, and improving the sensitivity of the piezoelectric acceleration sensor. The results show that the resonance frequency of the piezoelectric beam was 1072 Hz and the sensitivity of the proposed sensor was 2.05 V/g at the resonance frequency, which is 2.41-times higher than that of the piezoelectric beam. This research provides a good foundation for the integration of piezoelectric MOSFETs in the future. In future work, continuing to improve the sensitivity of piezoelectric acceleration sensors is an important research direction for us. We will improve the output sensitivity of the PMAS by optimizing the width-to-length ratio of MOSFETs and improving the manufacturing process of piezoelectric materials, while at the same time optimizing the cantilever beam structure to increase its frequency range.

## Figures and Tables

**Table 1:** Theoretical calculation parameters of resonance frequency.

*E* (GPa)	*ρ* (Kg/m^3^)	*π*	*l*_b_ (μm)	*w*_b_ (μm)	*h*_b_ (μm)	*l*_m_ (μm)	*w*_m_ (μm)	*h*_m_ (μm)
190	2330	3.14	6000	2400	80	1000	2700	395

**Table 2:** Performance comparison of the proposed sensor with others.

Transduction	Material	Die Area (mm^2^)	Sensitivity (V/g)	Reference
Piezoelectric	Li doped ZnO	9.8 × 5.8	2.05 at resonance frequency	this work
Piezoelectric	AlN	2.3 × 2.3	0.355	[38]
Piezoelectric	V doped ZnO	-	3.528 at resonance frequency	[25]
Piezoelectric	V doped ZnO	-	1.9 at resonance frequency	[39]
piezoresistive	Si	3.5 × 3.5	0.004	[40]
piezoresistive	Si	2 × 2	0.106	[41]
